# Preferential tau aggregation in von Economo neurons and fork cells in frontotemporal lobar degeneration with specific *MAPT* variants

**DOI:** 10.1186/s40478-019-0809-0

**Published:** 2019-10-22

**Authors:** Li-Chun Lin, Alissa L. Nana, Mackenzie Hepker, Ji-Hye Lee Hwang, Stephanie E. Gaus, Salvatore Spina, Celica G. Cosme, Li Gan, Lea T. Grinberg, Daniel H. Geschwind, Giovanni Coppola, Howard J. Rosen, Bruce L. Miller, William W. Seeley

**Affiliations:** 1 Memory and Aging Center, Weill Institute for Neurosciences, Department of Neurology, University of California, San Francisco, San Francisco, CA 94158 USA; 2 Gladstone Institute of Neurological Diseases, University of California, 1650 Owens St, San Francisco, CA 94158 USA; 3 Department of Pathology, University of California, 513 Parnassus Ave, San Francisco, CA 94143 USA; 4 Neurogenetics Program, Department of Neurology and Semel Institute for Neuroscience and Human Behavior, David Geffen School of Medicine, University of California Los Angeles, 760 Westwood Plaza, Los Angeles, CA 90095 USA

**Keywords:** Selective vulnerability, Tau, MAPT, von Economo neurons, Anterior cingulate cortex, Insula

## Abstract

Tau aggregation is a hallmark feature in a subset of patients with frontotemporal dementia (FTD). Early and selective loss of von Economo neurons (VENs) and fork cells within the frontoinsular (FI) and anterior cingulate cortices (ACC) is observed in patients with sporadic behavioral variant FTD (bvFTD) due to frontotemporal lobar degeneration (FTLD), including FTLD with tau inclusions (FTLD-tau). Recently, we further showed that these specialized neurons show preferential aggregation of TDP-43 in FTLD-TDP. Whether VENs and fork cells are prone to tau accumulation in FTLD-tau remains unclear, and no previous studies of these neurons have focused on patients with pathogenic variants in the gene encoding microtubule-associated protein tau (FTLD-tau/*MAPT*). Here, we examined regional profiles of tau aggregation and neurodegeneration in 40 brain regions in 8 patients with FTLD-tau/*MAPT* and 7 with Pick’s disease (PiD), a sporadic form of FTLD-tau that often presents with bvFTD. We further qualitatively assessed the cellular patterns of frontoinsular tau aggregation in FTLD-tau/*MAPT* using antibodies specific for tau hyperphosphorylation, acetylation, or conformational change. ACC and mid-insula were among the regions most affected by neurodegeneration and tau aggregation in FTLD-tau/*MAPT* and PiD. In these two forms of FTLD-tau, severity of regional neurodegeneration and tau protein aggregation were highly correlated across regions. In FTLD-tau/*MAPT*, VENs and fork cells showed disproportionate tau protein aggregation in patients with V337 M, A152T, and IVS10 + 16 variants, but not in patients with the P301L variant. As seen in FTLD-TDP, our data suggest that VENs and fork cells represent preferentially vulnerable neuron types in most, but not all of the *MAPT* variants we studied.

## Introduction

The frontoinsula (FI) and anterior cingulate cortex (ACC) are key hubs within a large-scale “salience network” critical for autonomic and social-emotional functions [1, 2]. These regions are the earliest and most consistently affected in patients with sporadic behavioral variant frontotemporal dementia (bvFTD) [3–5] and represent the major sites where von Economo neurons (VENs) and fork cells are located. Early, selective loss of these unique Layer 5 neurons has been demonstrated in patients with sporadic bvFTD across the underlying FTLD spectrum, including patients with tau-immunoreactive inclusions (FTLD-tau) [6–11]. In FTLD with transactive response DNA binding protein 43 kDa (TDP-43) inclusions (FTLD-TDP), VENs show a striking propensity to form TDP-43 inclusions [12], but whether the same is true for tau aggregation in FTLD-tau remains unstudied. Pick’s disease (PiD) is the most common FTLD-tau subtype underlying bvFTD, but its severe neuronal loss makes it difficult to study early neuronal targets of tau aggregation. Given the growing efforts to model FTLD tauopathy using cell models derived from patients with FTLD-tau/*MAPT* [13–15], we reasoned that an assessment of VEN and fork cell vulnerability in this patient group could provide an important backdrop for the field. We hypothesized that ACC and FI VENs and fork cells are prone to tau aggregation in FTLD-tau, as they are to TDP-43 aggregation in FTLD-TDP. Here, we combined a semi-quantitative regional analysis with a qualitative assessment of neuron type-specific aggregation of tau in ACC and FI. Forty brain regions were prospectively rated for neurodegeneration and tau inclusions in 8 patients with FTLD-tau/*MAPT* and 7 with sporadic bvFTD-PiD. We then focused on tau aggregation within VENs, fork cells, and neighboring neurons in patients with FTLD-tau/*MAPT* representing four *MAPT* variants from different exons, introns, and families (V337 M: exon 12 mutation; P301L: exon 10 mutation; IVS10 + 16: intron 10 mutation; A152T: risk variant), using monoclonal antibodies labeling tau acetylation, hyperphosphorylation, and conformational changes. The findings suggest overlapping regional and neuron type-specific vulnerability in sporadic and inherited FTLD-tau.

## Material and methods

### Patients and autopsy procedures

Post-mortem human brain tissue was obtained from the UCSF Neurodegenerative Disease Brain Bank. Clinical diagnoses of bvFTD, non-fluent variant primary progressive aphasia (nfvPPA), and progressive supranuclear palsy-Richardson syndrome (PSP-RS), were made according to prevailing international consensus criteria at the time of assessment [16, 17]. Neuropathological diagnoses were made following consensus diagnostic criteria using previously described histological and immunohistochemical methods [18–20]. Cases were selected based on clinical and neuropathological diagnoses, and genetic analysis [7, 21] (Table 1 Additional file 1: Table S1). Initial brain cutting and processing depended on the site of brain procurement. For Cases 1–4, 6–8 of FTLD-tau/*MAPT* group with Case 3 of PiD group, one cerebral hemisphere was immersion fixed in 10% buffered formalin indefinitely. The remaining cases were cut freshly into ~ 1 cm-thick coronal slabs and fixed in 10% neutral buffered formalin for ~ 72 h. The FTLD-tau/*MAPT* cohort (*n* = 8) consisted of P301L (exon 10; *n* = 2, relatives), IVS10 + 16 (intron 10; *n* = 3, relatives), V337 M (exon 12, *n* = 1), and A152T (n = 2, unrelated families). Regional involvement in FTLD-tau/*MAPT* was compared to sporadic bvFTD due to PiD (*n* = 7).

**Table 1:** Subject demographics

Case no.	Clinical Dx	Primary Neuropathological Dx	*MAPT* variant	Age/Sex	Symptom duration (yr)	PMI (hr)	Thal phase	Braak stage	CERAD score	ADNC	CDR score
FTLD-tau/*MAPT*											
1	bvFTD/PSP-RS	FTLD-tau/*MAPT*	IVS10 + 16 C > T	60/M	8	12.5	ND	1	absent	Low	2^a^
2	bvFTD	FTLD-tau/*MAPT*	IVS10 + 16 C > T	60/M	13	15.8	1	0	absent	Low	3^b^
3	bvFTD	FTLD-tau/*MAPT*	IVS10 + 16 C > T	63/F	17	19.7	1	0	absent	Low	3
4	bvFTD	FTLD-tau/*MAPT*	P301L	59/F	4	24.2	1	1	absent	Low	2
5	bvFTD	FTLD-tau/*MAPT*	P301L	75/M	9	8.6	1	2	absent	Low	3
6	bvFTD	FTLD-tau/*MAPT*	V337 M	68/F	13	18.0	0	1	absent	Not	3
7	PSP-RS	PSP	A152T	56/F	6	5.7	2	2	absent	Low	3
8	nfvPPA	CBD	A152T	63/M	10	> 48 h	1	3	absent	Low	1
Average				63	10	15	1	1			2.5
Sporadic bvFTD-PiD											
1	bvFTD	PiD	None	60/F	6	15.1	2–3	1	moderate	Low	3^c^
2	bvFTD	PiD	None	57/M	6	21.7	0	0	absent	Not	2^d^
3	bvFTD	PiD	None	70/M	10	10.2	2	0	moderate	Low	n/a
4	bvFTD	PiD	None	72/M	9	24.3	0	0	absent	Not	3
5	bvFTD	PiD	None	69/F	13	6.3	0	2	absent	Not	3
6	bvFTD	PiD	None	76/F	13	25.5	0	0	absent	Not	3
7	bvFTD	PiD	None	57/M	13	12.4	1	1	absent	Low	3
Average				66	10	17	1	1			2.8

^a^antemortem, 3 months prior to death, ^b^antemortem, 20 months prior to death, ^c^antemortem, 18 months prior to death, ^d^antemortem, 7 months prior to death, diagnosis (Dx), behvaioral variant FTD (bvFTD), progressive supranuclear palsy-Richardson syndrome (PSP-RS), nonfluent variant primary progressive aphasia (nfvPPA), corticobasal degeneration (CBD), Pick’s disease (PiD), Postmortem interval (PMI)

### Immunohistochemistry

Twenty-five standard diagnostic fixed paraffin-embedded tissue blocks, encompassing 40 distinct brain regions for each brain, were cut from one hemisphere into 8 μm-thick sections, mounted on glass slides, deparaffinized, and stained. For phospho-tau immunostaining, paraffin sections underwent heat-induced antigen retrieval using an autoclave at 121 °C in citrate buffer, pH 6.0 for 5 min. Sections were then incubated with an established antibody CP13 (mouse monoclonal, targeting pSer202, gift from Peter Davies [22]) overnight at room temperature. Following incubation with CP13, sections were next incubated at room temperature for 40 min with biotinylated secondary antibody (1200, Vector Laboratories) before incubation for 30 min with avidin-biotin-peroxidase complexes (1100, VECTASTAIN Elite Kit, PK-6100, Vector Laboratories). Staining was developed using the chromogen 3,3-diaminobenzidine tetrahydrochloride (DAB; Fisher) /H_2_O_2_ and sections were counterstained with Hematoxylin before coverslipping in Permaslip (Alban Scientific).

For free-floating immunohistochemistry, tissue blocks from ACC and FI were dissected from ~ 1 cm thick formalin-fixed coronal slabs, immersed in graded sucrose solutions (10, 20, 30% sucrose in PBS with sodium azide), and sectioned on a sliding microtome into alternating series of 300- and 50 μm- sections. Every 12th section was Nissl-stained with cresyl violet (FD NeuroTechnologies) to determine the anatomical boundaries of the region of interest. Three sections from each block were stained for tau hyperphosphorylation (CP13), acetylation (MAB359; rabbit monoclonal, targeting K274, gift from Li Gan [23]), or conformational changes (MC1; mouse monoclonal, gift from Peter Davies [24]). Sections were thoroughly rinsed in 0.01 M PBS (6 × 10 min). MAB359-stained sections were pre-treated with 88% formic acid for 5 min and then underwent antigen retrieval in 10 mM citrate buffer, pH 6.0 for 5 min at 121 °C. MC1-stained sections underwent antigen retrieval in 10 mM Tris buffer at pH 9.0 for two hours at 80 °C. CP13-stained sections underwent antigen retrieval in 10 mM citrate buffer pH 6.0 for two hours at 80 °C. After washing with PBS (3 × 10 min), CP13- and MC- stained sections were incubated in 3% H_2_O_2_ diluted in PBS-Az for 30 min to block endogenous peroxidase activity. Sections were then washed and incubated in 0.01 M PBS containing 0.3% Triton X-100 and 10% normal horse or goat serum (Vector Laboratories, Burlingame, CA, USA) for 1 h, followed by incubation with CP-13 (1:5000), MAB359 (1:10,000), or MC1 (1:500) in 0.01 M PBS containing 0.3% Triton X-100, 10% normal serum for 48 h at 4 °C. Sections were then incubated with biotinylated secondary antibody (horse anti-mouse or goat anti-rabbit IgG; Vector Laboratories, Burlingame, CA; 1:500) in antibody buffer for one hour at room temperature. Sections were then washed and incubated with avidin-biotin-peroxidase complexes (Vectastain Elite Kit, PK-6100, Vector Laboratories, 1:500 each in PBT) for one hour. After washing, immunostaining was developed with DAB. Selected sections were counterstained with thionin (PS101–02; FD NeuroTechnologies). A Nikon 80i microscope was used for bright-field microscopy. Photomicrographs were taken using a Nikon Digital Sight DS-Fi1 camera and NIS Elements D 3.2 software.

### Neuropathological evaluation

For regional semi-quantitaive analysis, we performed routine immunohistochemistry and assessments with a single 8 μm-thick section from each standard diagnostic fixed paraffin-embedded tissue block. For cellular qualitative analysis, we performed free-floating immunohistochemistry and assessments with three 50 μm-thick sections equally spaced throughout each ACC and FI tissue block. Stained sections from a standard set of brain regions were prospectively assessed by one of three trained examiners (WWS, LTG, or SS). Pathological diagnostic, staging, and semi-quantitative regional neuropathological data were performed by one of three expert examiners (WWS, LTG, or SS) and reviewed at a consensus case conference. The assessments were performed prospectively without knowledge of the study hypotheses but were not blinded to clinical or genetic information. Examiners meet regularly to promote reliability of these prospective diagnostic assessments. Nonspecific features of neurodegeneration were scored based on the hematoxylin and eosin (H&E) stain and included microvacuolation, astrogliosis, and neuronal loss, each graded on a 0 to 3 scale (absent, mild, moderate, severe). Tau aggregates were visualized based on CP13 staining and rated using the same 0–3 scale for neurofibrillary tangles, Pick bodies, (other) neuronal cytoplasmic inclusions, globose tangles, astrocytic plaques, tufted astrocytes, thorny astrocytes, tau-positive threads and grains in the gray and white matter, and (other) glial cytoplasmic inclusions. We generated neurodegeneration scores for each region in each case by adding the score of “neuronal loss” to the average of the scores for “vacuolation” and “gliosis” based on the H&E stain for each region in each case. The tau scores were the average scores across “neurofibrillary tangles,” “Pick bodies,” “(other) neuronal cytoplasmic inclusions,” “globose tangles,” “astrocytic plaques,” “tufted astrocytes,” “thorn-shaped astrocytes,” “tau-positive threads,” and grains in the gray and white matter, and (other) glial cytoplasmic inclusions. To analyze the overall pattern pathological changes, we calculated a composite score for each region by adding the tau and neurodegeneration scores; we then averaged composite scores across subjects for each region. The median composite score was used to rank order brain regions within each patient group. For patients with FTLD-tau/*MAPT*, thick sections submitted for immunohistochemistry were assessed qualitatively to determine the pattern of VEN vs. neighboring neuron tau aggregation propensity. PiD cases showed advanced stages of disease with massive loss of VENs and fork cells, making them unsuitable for assessment of tau aggregation at the cellular level.

### Statistical analysis

Pearson’s correlation tests for linear regression were performed using GraphPad Prism 8 to assess the associations between neurodegeneration and tau aggregation scores across each brain region in FTLD-tau/*MAPT* and FTLD-PiD. We employed a *p* < 0.05 (two-tailed) threshold for a statistical significance.

## Results

### FTLD-tau/*MAPT* and PiD share prominent involvement of ACC and mid-insular cortex

**Fig. 1:**
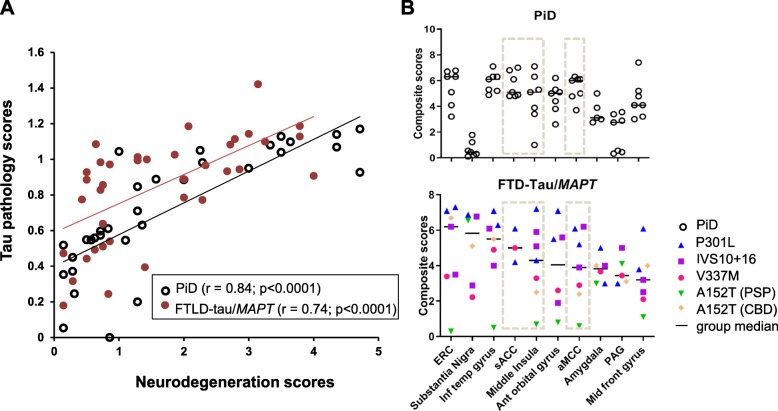
**a** Correlation of neurodegeneration and tau pathology in the FTLD-tau/*MAPT* and PiD cases across regions. **b** Ten most affected regions in the FTLD-tau/*MAPT* cases showed similar ranking of regional burden as PiD cases. The insular and cingulate cortex are highlighted between dashed lines

To evaluate how our patients with FTLD-tau/*MAPT* (*n* = 8) compared to patients with bvFTD due to sporadic PiD (*n* = 7), we assessed regional neurodegeneration (ND) and tau aggregation in 40 brain regions per case. As expected, we found a strong positive correlation between ND and tau aggregation across brain regions in FTLD-tau/*MAPT* (r = 0.74, *p* < 0.0001) and in PiD (r = 0.84, *p* < 0.0001) (Fig. 1a). The pattern of regional involvement also suggested a substantial overlap between groups, including severe degeneration in the pre- and sub-genual ACC and mid-insular cortex. Notably, tau burden in FTLD-tau/*MAPT* and PiD reached a plateau in the presence of severe neurodegeneration. As in PiD, in FTLD-tau/*MAPT* the ACC subregions and insula were among the ten most affected regions, based on the regional composite scores (Fig. 1b). In addition to ACC and middle insula, amygdala and PAG, also nodes within the salience network, were among the 10 most affected regions in FTLD-tau/*MAPT*. The other 7 most-affected regions for FTLD-tau/*MAPT* were included in the top 10 for PiD. Overall, the FTLD-tau/*MAPT* cases had a similar ranking of regional burden to the PiD cases*.* P301L cases showed the most severe tau burden, with relatively high composite scores across most regions, including those in the salience network, followed by IVS10 + 16 cases, then V337 M. As expected, the A152T case with underlying PSP, which primarily affects subcortical and brainstem structures, showed the mildest tau deposition in cortical regions.

### VENs and fork cells in most FTLD-tau/*MAPT* variants show disproportionate tau aggregation featuring phosphorylation and acetylation

**Fig. 2:**
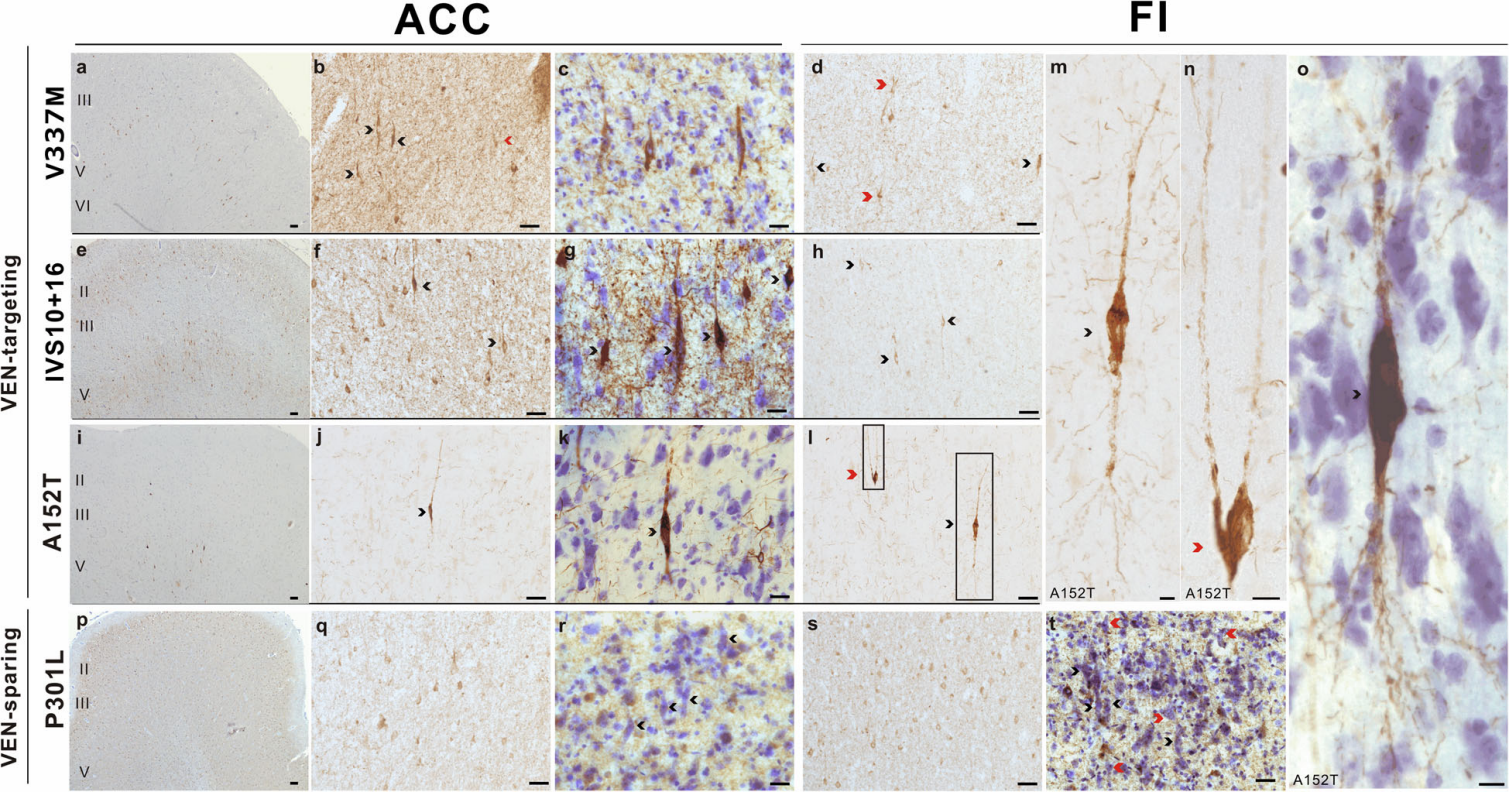
VENs (black arrowheads) and fork cells (red arrowheads) showed a high rate of hyperphosphorylated tau inclusion formation compared to neighboring neurons in Layer 5 in patients with V337 M (**a**-**d**), IVS10 + 16 (**e**-**h**), A152T (**i**-**o**); but not the P301L variant (**p**-**t**). Scale bars: a, e, i, *p* = 100 μm; b, d, f, h, j, l, q-t = 50 μm; c, g, k = 25 μm; m-o = 10 μm

Having confirmed that the ACC and mid-insula were both prominently affected in FTLD-tau/*MAPT*, we next focused on whether ACC and FI VENs and fork cells [25, 26] showed an increased propensity to aggregate tau compared to neighboring neurons. This hypothesis of selective neuronal vulnerability was supported by the pattern of tau hyperphosphorylation in the patients with the V337 M variant (Fig. 2a-d) and A152T variant (Fig. 2i-o), who showed neurofibrillary tangle-like cytoplasmic inclusions, all 3 patients with the IVS10 + 16 variant (Fig. 2e-h), who showed a more diffuse/granular inclusion type, but not in the two patients with the P301L variant (Fig. 2p-t). All of these patients presented with bvFTD. The *MAPT* A152T rare variant is considered a risk factor for tauopathies including FTLD-tau and AD [27], and we included both of the patients with this variant available within the UCSF Neurodegenerative Disease Brain Bank. Staining for hyperphosphorylated tau revealed that tau aggregates typically filled the VEN and fork cell somata and continued into proximal apical and basal dendrites in the FTLD-PSP/A152T case (Fig. 2m-o). Despite the sparse tau aggregation in the ACC and insula, VENs and fork cells were clearly among the most, if not the most vulnerable to tau hyperphosphorylation (Fig. 2), representing a higher proportion of inclusion-bearing neurons than predicted by their low prevalence within Layer 5 (2–5% in control brains, depending on region and subregion) [28]. The patterns of tau aggregation differed between the two patients with the A152T variant, not surprisingly given that one had PSP and the other CBD. The most affected regions in the FTLD-PSP/A152T case were the subcortical regions involved in motor function, including dentate nucleus, tectum, substantia nigra, global pallidus, as typically seen in patients with Richardson syndrome due to PSP. Thus, the FTLD-PSP/A152T case provided valuable information about the most vulnerable cortical neuron populations at an early stage of tau aggregation (Fig. 2i-o). The patients with FTLD-CBD/A152T presented with non-fluent variant primary progressive aphasia at an advanced stage. The cortical regions, including ERC, posterior cingulate cortex, middle frontal gyrus, inferior temporal gyrus, middle insula, angular gyrus, and anterior midcingulate cortex, were most affected. Most hyperphosphorylated tau labeling in the CBD/A152T case was seen in threads and neuronal cytoplasmic inclusions in the ACC, and a few labeled neuronal cytoplasmic inclusions in the FI. In contrast, the two patients with the P301L variant showed a heavy tau burden more prominent in Layer 6 than 5, with conspicuous sparing of VENs and fork cells (Fig. 2r, t-p) despite a clinical presentation of bvFTD.

**Fig. 3:**
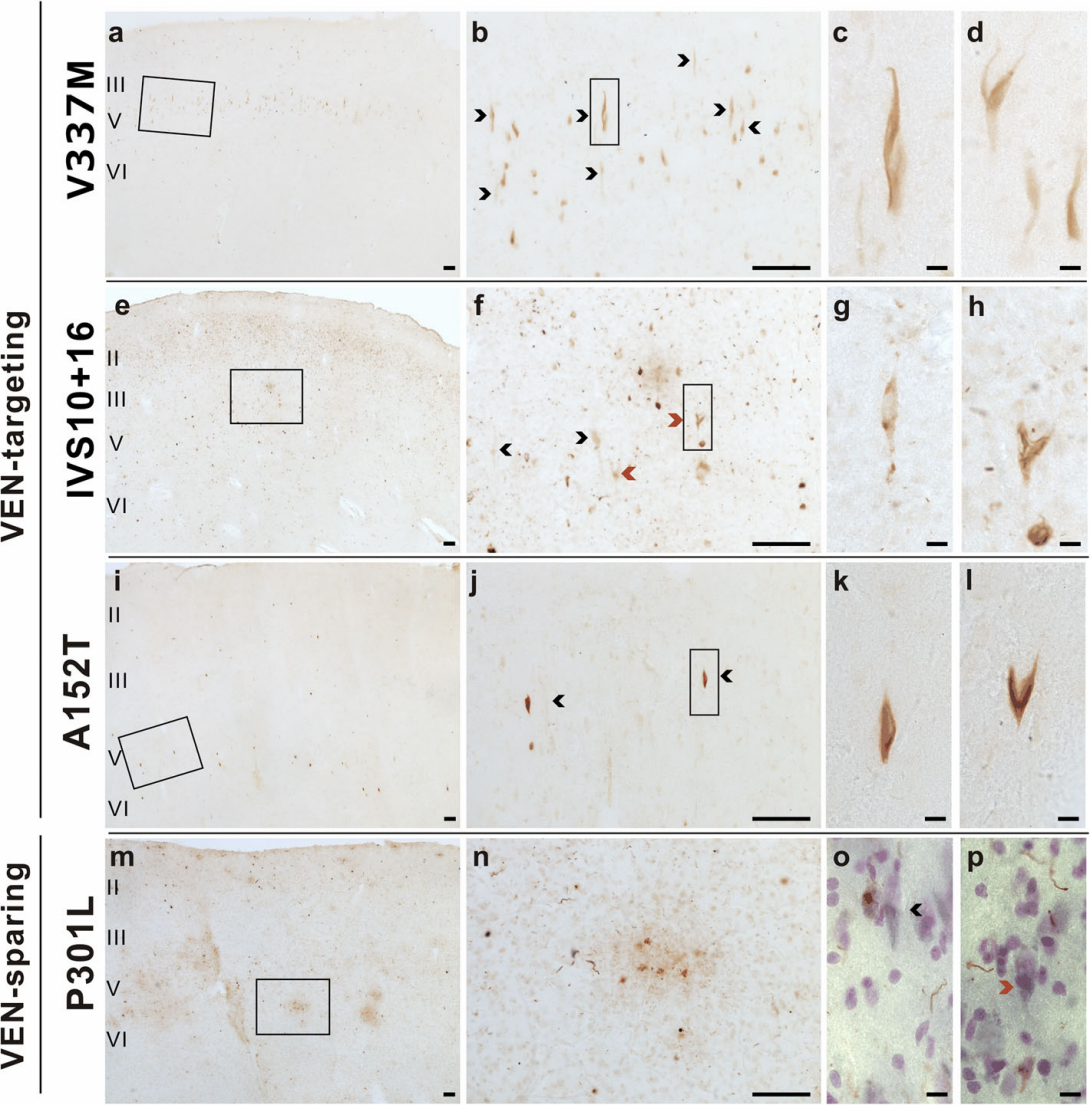
VENs (black arrowheads) and fork cells (red arrowheads) showed a high rate of acetylated tau inclusion formation compared to neighboring Layer 5 neurons in patients with V337 M (**a**-**d**), IVS10 + 16 (**e**-**h**), PSP/A152T (**i**-**l**), but not the P301L variant (**m**-**p**). Scale bars: a, b, e, f, i, j, m, *n* = 100 μm; c, d, g, h, k, l, o, *p* = 10 μm

The dense burden of neuropil tau hyperphosphorylation seen in some patients and regions precluded clear inferences about relative cell-type vulnerabilities. Therefore, we further employed an antibody to tau acetylated at K274, which generally labels acetylated tau contained within the proximal neuron but not more distal processes that compose the neuropil. This strategy allowed us to assess tau acetylation in VENs and fork cells while also gaining a clearer picture of cell type specificity (Fig. 3).

The identified VENs and fork cell inclusions adopted a neurofibrillary character consistent with the six-isoform nature of the V337 M tauopathy (Figs. 2 and 3). In the patient with the V337 M variant (Fig. 3a-d), tau acetylation was predominantly in Layer 5 (Fig. 3a-b), and a qualitative survey revealed most of these were VENs and fork cells. In IVS10 + 16 cases, the tau aggregates in VENs and fork cells were less fibrillary, forming more diffuse, patchy and granular material within the cytoplasm (Fig. 3e-h). Grains were copious in the ventral (agranular, VEN/fork cell-containing) anterior insula but sparse in dorsal (dysgranular, VEN/fork cell-lacking) anterior insula in all 3 patients with the IVS10 + 16 variant, all of whom shared a common tau deposition pattern, with abundant acetylated and hyperphosphorylated tau grains in the superficial layers and relatively few acetylated tau-positive neuronal cytoplasmic inclusions in superficial or deep layers. VENs and fork cells were clearly represented among the few inclusion-bearing neurons, despite their low prevalence in the tissue, indicating a predisposition toward inclusion formation in the IVS10 + 16 variant. Acetylated tau-positive neuronal cytoplasmic inclusions in the PSP/A152T case (Fig. 3i-l) showed a pattern similar to but less frequent than of the stains for tau hyperphosphorylation (Fig. 2i-o). As previously shown in CBD [23], most acetylated tau labeling in the CBD/A152T case was seen in astrocytic plaques with few labeled neuronal cytoplasmic inclusions. In the P301L cases, acetylated tau findings mirrored those seen with hyperphosphorylated tau. VEN and fork cell numbers appeared relatively normal, and those cells identified in Nissl-counterstained materials rarely showed tau acetylation (Fig. 3m-p), despite severe degeneration in the ACC and mid-insula (Fig. 1b).

### VENs and fork cells in most FTLD-tau/MAPT variants show conformational changes of tau

**Fig. 4:**
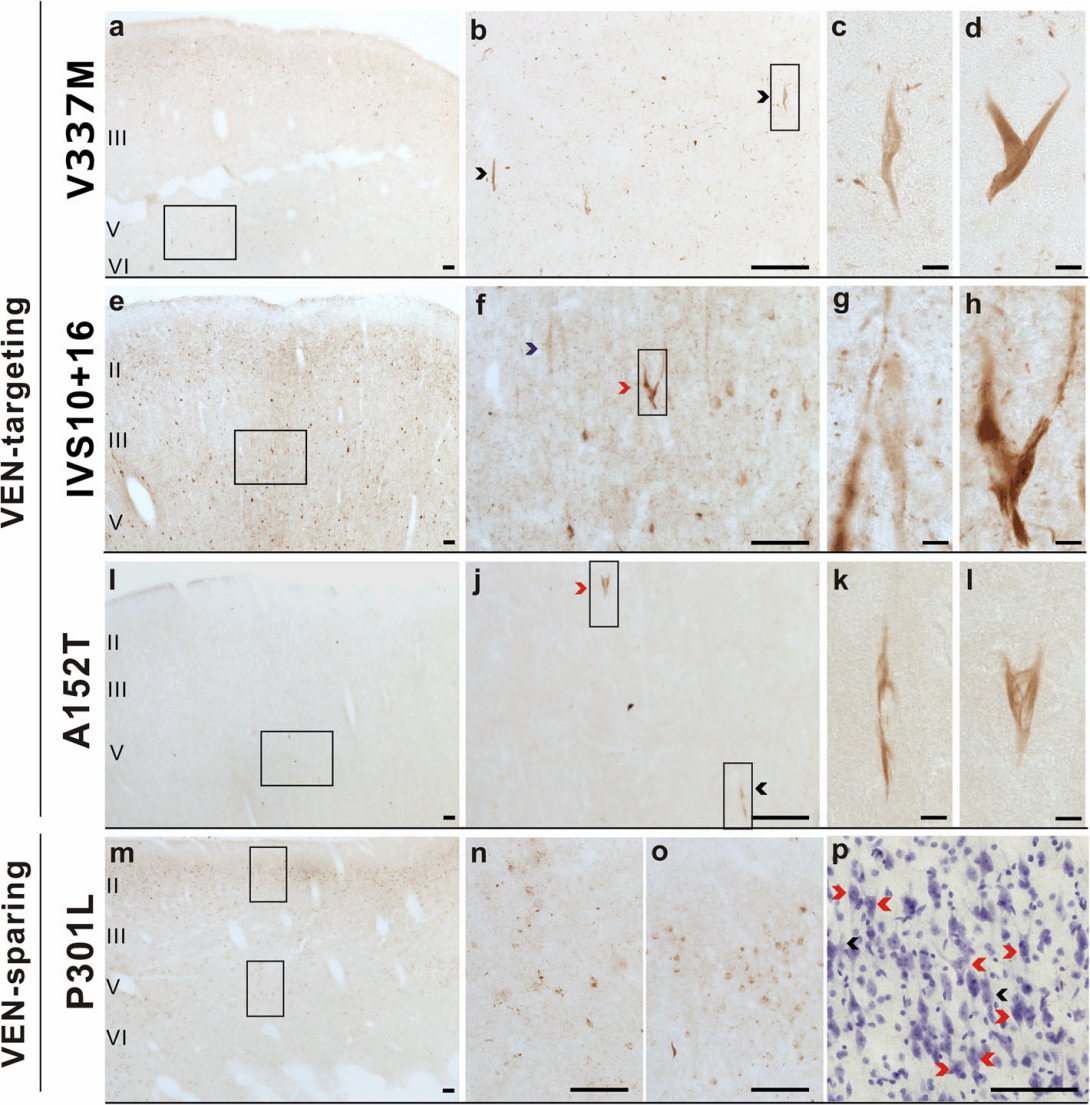
VENs (black arrowheads) and fork cells (red arrowheads) showed a high rate of conformational changes of tau compared to neighboring Layer 5 neurons in patients with V337 M (**a**-**d**), IVS10 + 16 (**e**-**h**), and A152T (**i**-**l**), but not the P301L variant (**m**-**p**). Scale bars: a, b, e, f, i, j, m, n, o, p = 100 μm; c, d, g, h, k, l = 10 μm

Conformational changes in the tau protein modify its function [29, 30]. To test whether VENs and fork cells show tau conformational changes in FTLD-tau/*MAPT*, we used a conformation-specific monoclonal antibody (MC1), which detects a discontinuous, conformational epitope of tau protein at amino acid residues 7–9 and 312–322 in the third microtubule binding domain. In V337 M, MC1-positive inclusions were very sparse, but VENs and fork cells were prominent among the labeled neurons (Fig. 4a-d). The IVS10 + 16 and A152T cases demonstrated formation of pre-tangle-like inclusions in VENs and fork cells, affecting only a subset of the tau inclusion-bearing cells expected based on other staining methods (Fig. 4e-l). In the P301L cases, VENs and fork cells were again conspicuously spared despite widespread MC-1 staining in general (Fig. 4m-p). We found higher rates of MC1 immunoreactivity in IVS10 + 16 and P301L, followed by V337 M and lastly the A152T cases.

Taken together, our findings suggest that VENs and fork cells are predisposed to tau aggregation in FTLD-tau/*MAPT* with V337 M, IVS + 16, and A152T variants but may be less vulnerable, in patients with the P301L variant (Table 2).

**Table 2:** Summary of pathological tau inclusion patterns in selected patients with FTLD-tau/*MAPT*

Pathological changes in FTLD-tau/*MAPT*	IVS10 + 16 C- > T	V337 M	A152T	P301L
Cortical Layer NCI distribution	2, 5–6	2, 5–6	5 (PSP)	2, 5–6
Preferential VEN and fork cell tau aggregation	Yes	Yes	Yes	No
Hyperphosphorylation at serine 202 (CP13)	Yes	Yes	Yes	Yes
Acetylation at k274 (MAB359)	Moderate	Mild	Mild	Mild
Tau conformational change in NCIs (MC1)	Mild-moderate	Mild	Absent-Mild	Moderate-Severe

## Discussion

Sporadic and familial bvFTD are associated with neurodegeneration that either begins in or quickly spreads to the ACC and FI [4, 31–33]. Targeting of these regions has been linked to selective dropout of VENs and fork cells [6–10, 12], but to date no study has assessed tau inclusion formation within these neurons. Here, we used a qualitative approach to show that bvFTD due to inherited FTLD-tau, like sporadic bvFTD due to PiD, shows an anatomical pattern that prominently includes the ACC and FI. We focused our VEN/fork cell assessment on FTLD-tau/*MAPT* because the VEN-containing regions in PiD were too degenerate to enable a conclusive anatomical assessment. Our FTLD-tau/*MAPT* findings showed frequent tau inclusions in VENs and fork cells, out of proportion to the abundance of these neurons in the tissue, in patients with the V337 M, IVS + 16, and A152T variants (Table 2). Findings from the FTLD-PSP/A152T case suggested that VENs and fork cells were affected during the early stages of cortical involvement, at a time when Layer 2–3 neurons are just beginning to form tau inclusions and neighboring Layer 5 neurons remain largely spared. Intriguingly, our two patients with the P301L variant showed conspicuous sparing of these neurons. Although these findings should be viewed with caution in light of the small sample sizes within each *MAPT* variant, our observations suggest that VENs and fork cells are vulnerable to tau aggregation in FTLD-tau/*MAPT*. This vulnerability may interact with the specific *MAPT* variant in ways that remain to be explored.

There are several potential implications of this study. First, our findings suggest the possibility that *MAPT* variants, and their consequent changes in tau protein structure and function, direct the pattern of cell type vulnerability to tau aggregation. This observation presents an opportunity to explore the interaction between misfolded tau structures (or “strains”) and neuron type in determining where disease begins and spreads. Second, VEN and fork cell degeneration in ACC and FI is strongly linked to the bvFTD syndrome, but in the present study bvFTD emerged in patients with and without targeted VEN/fork cell tau aggregation. This observation suggests that phenotypic convergence need not be determined at the neuron type level and may instead be driven, in some patients, by neuroanatomical convergence at the regional or network levels. Compared to other *MAPT* variants, patients with P301L variant showed relatively high composite scores in the regions linked to the salience network, including sACC, middle insula, aMCC, and amygdala. Thus, in some cases, such as P301L, neuroanatomical convergence producing the bvFTD syndrome may occur at the regional or network level. In other words, dysfunction within ACC, FI, or their salience network partners may prove sufficient to drive social-emotional dysfunction in bvFTD even when VENs and fork cells remain intact, as observed in our *MAPT* P301L variant carriers. Third, our findings suggest that cell-based assays designed to model selective vulnerability in vitro may require tailoring to the individual disease-causing or risk variant and its established selective vulnerability pattern. Fourth, our findings lend additional support for ongoing efforts to differentiate induced pluripotent stem cells and fibroblasts into a VEN and fork cell lineage, parallel to protocols that derive motor neuron-like cells for the study of amyotrophic lateral sclerosis or dopaminergic neurons for the study of Lewy body disease.

### Limitations and future directions

This study was limited by materials available at the UCSF Neurodegenerative Disease Brain Bank, which resulted in small samples within each *MAPT* variant, too small to afford statistical comparisons. Based on these constraints and the visually striking effects observed in the tissues, we opted for a qualitative survey of VEN and fork cell tau aggregation. The stage is set for future larger studies with more *MAPT* variants and a quantitative approach to neuron-type vulnerability assessment. The three IVS10 + 16 cases had relatively severe neuronal loss, possibly undermining our ability to detect VENs and fork cells containing tau inclusions. The MC1 antibody used to assess conformational changes in tau was developed for Alzheimer’s disease and comparable reagents for these diverse inherited tauopathies are lacking [34]. Despite these limitations, our findings provide a foundation for future exploration of selective vulnerability in FTLD-tau/MAPT and other FTLD tauopathies.

## Conclusions

The available data suggest that some *MAPT* variants may converge on these large, specialized neurons through commonalities among their misfolded tau “strains”, by disrupting some common cellular process critical for VEN/fork cell survival, or through independent, variant-specific mechanisms that await exploration.

## Supplementary information

**Additional file 1: Table S1.** Alzheimer’s disease-related changes in patients with FTLD-tau/*MAPT* and Pick’s disease.

## Data Availability

The datasets used and/or analysed during the current study available from the corresponding author on reasonable request.

## Abbreviations

ACC: Anterior cingulate cortex
aMCC: anterior midcingulate cortex
bvFTD: behavioral variant FTD
DAB: Diaminobenzidine
ERC: Entorhinal cortex
FI: Frontoinsula
FTD: Frontotemporal dementia
FTDP-17: Frontotemporal dementia with parkinsonism-17
FTLD: Frontotemporal lobar degeneration
FTLD-PiD: FTLD patients with Pick’s disease
FTLD-tau/*MAPT*: FTLD with pathogenic variants of microtubule-associated protein tau
H&E: Hematoxylin and eosin
PAG: Periaqueductal gray
sACC: subgenual anterior cingulate cortex
VENs: Von Economo neurons
