# Supplementary material for: Preferential tau aggregation in von Economo neurons and fork cells in frontotemporal lobar degeneration with specific MAPT variants
Source: Acta Neuropathol Commun. 2019 Oct 22;7:159. doi: 10.1186/s40478-019-0809-0 (PMC6805408; doi:10.1186/s40478-019-0809-0)
Supplement: Supplementary file 1 — Additional file 1: Table S1. Alzheimer’s disease-related changes in patients with FTLD-tau/MAPT and Pick’s disease. [file 40478_2019_809_MOESM1_ESM.docx]

**Additional file 1 Table S1. Alzheimer’s disease-related changes in patients with FTLD-tau/*MAPT* and Pick’s disease**

| **Case no.** | **Clinical Dx** | **Neuropath Dx** | ***MAPT* variant** | **ADNC** | **Thal Amyloid Plaque Phase** | **Braak Stage** | **CERAD** | **Additional pathological changes** |
| --- | --- | --- | --- | --- | --- | --- | --- | --- |
| 1 | bvFTD/PSP-RS | FTLD-tau/*MAPT* | IVS10+16 C>T | Low | ? | 1 | absent | None |
| 2 | bvFTD | FTLD-tau/*MAPT* | IVS10+16 C>T | Low | 1 | 0 | absent | Lewy body disease, brainstem only type |
| 3 | bvFTD | FTLD-tau/*MAPT* | IVS10+16 C>T | Low | 1 | 0 | absent | None |
| 4 | bvFTD | FTLD-tau/*MAPT* | P301L | Low | 1 | 1 | absent | None |
| 5 | bvFTD | FTLD-tau/*MAPT* | P301L | Low | 1 | 2 | absent | limbic TDP-43 proteinopathy |
| 6 | bvFTD | FTLD-tau/*MAPT* | V337M | Not | 0 | 1 | absent | Lewy body disease, transitional limbic type |
| 7 | PSP-RS | PSP | A152T | Low | 2 | 2 | absent | None |
| 8 | nfvPPA | CBD | A152T | Low | 1 | 3 | absent | TDP-43 proteinopathy; Argyrophilic grain disease; Lewy body disease (limited to substantia nigra, locus ceruleus and amygdala) |
|  |  |  |  |  |  |  |  |  |
| **Case no.** | **Clinical Dx** | **Neuropath Dx** | ***MAPT* variant** | **ADNC** | **Thal Amyloid Plaque Phase** | **Braak Stage** | **CERAD** | **Additional pathological changes** |
| 1 | bvFTD | PiD | None | Low | 2-3 | 1 | moderate | None |
| 2 | bvFTD | PiD | None | Not | 0 | 0 | absent | None |
| 3 | bvFTD | PiD | None | Low | 2 | 0 | moderate | None |
| 4 | bvFTD | PiD | None | Not | 0 | 0 | absent | None |
| 5 | bvFTD | PiD | None | Not | 0 | 2 | absent | None |
| 6 | bvFTD | PiD | None | Not | 0 | 0 | absent | Vascular brain injury |
| 7 | bvFTD | PiD | None | Low | 1 | 1 | absent | None |
